# Potential interplay between tumor size and vitamin D receptor (*VDR*) polymorphisms in breast cancer prognosis: a prospective cohort study

**DOI:** 10.1007/s10552-023-01845-1

**Published:** 2024-02-14

**Authors:** Hampus Lindgren, David Ademi, Christopher Godina, Helga Tryggvadottir, Karolin Isaksson, Helena Jernström

**Affiliations:** 1 Division of Oncology, Department of Clinical Sciences, Lund, Lund University and Skåne University Hospital, Barngatan 4, SE 221 85 Lund, Sweden; 2 Division of Surgery, Department of Clinical Sciences, Lund, Lund University, SE 221 85 Lund, Sweden; 3 Department of Surgery, Kristianstad Hospital, J A Hedlunds väg 5, SE 291 33 Kristianstad, Sweden

**Keywords:** Breast cancer, Vitamin D receptor (VDR) polymoprhisms, Prognosis, Population-based cohort

## Abstract

**Purpose:**

Vitamin D has some anticancer properties that may decrease breast cancer risk and improve prognosis. The aim was to investigate associations between four previously studied *VDR* SNPs (Taq1, Tru91, Bsm1, and Fok1) and prognosis in different groups of breast cancer patients.

**Methods:**

*VDR* genotyping of 1,017 breast cancer patients included 2002–2012 in Lund, Sweden, was performed using Oncoarray. Follow-up was until June 30, 2019. Clinical data and patient information were collected from medical records and questionnaires. Cox regression was used for survival analyses.

**Results:**

Genotype frequencies were as follows: Fok1 (AA 15.7%, AG 49.1%, GG 35.1%), Bsm1 (CC 37.2%, CT 46.1%, TT 16.7%), Tru91 (CC 77.8%, CT 20.7%, TT 1.5%), and Taq1 (AA 37.2%, AG 46.2%, GG 16.6%). During follow-up there were 195 breast cancer events. The homozygous variants of Taq1 and Bsm1 were associated with reduced risk of breast cancer events (adjusted HR = 0.59, 95% CI 0.38–0.92 for Taq1 and adjusted HR = 0.61, 95% CI 0.40–0.94 for Bsm1). The G allele of the Fok1 was associated with increased risk of breast cancer events in small tumors (pT1, adjusted HR = 1.83, 95% CI 1.04–3.23) but not in large tumors (pT2/3/4, adjusted HR = 0.80, 95% CI 0.41–1.59) with a borderline interaction (*P*_interaction_ = 0.058). No interactions between *VDR* genotypes and adjuvant treatments regarding breast cancer prognosis were detected.

**Conclusion:**

VDR genotypes were associated with breast cancer prognosis and the association might be modified by tumor size. Further research is needed to confirm the findings and elucidate their potential clinical implications.

**Supplementary Information:**

The online version contains supplementary material available at 10.1007/s10552-023-01845-1.

## Introduction

Breast cancer is the most prevalent form of cancer among women in Sweden and globally, accounting for 29.5% and 24.5%, respectively, of all new cancer cases among women in 2020 [1, 2]. Despite a rise in incidence over the recent decades in Sweden, breast cancer mortality has decreased and in 2019 the 10-year survival was 87.1% [3]. The prognosis differs significantly depending on patient characteristics, such as age, and tumor characteristics, including estrogen receptor (ER) status [4]. Therefore, there is still a need for a more personalized selection of adjuvant treatments, as well as new treatment options. Vitamin D and its receptor (VDR) have in some studies been shown to counteract tumor progression and enhance different treatments, which merits further investigation [5].

Vitamin D has several biological functions throughout the body including its first discovered function of regulating bone metabolism [6, 7]. Further, vitamin D has been shown to induce apoptosis [8], inhibit proliferation [9, 10], induce differentiation in a variety of cancer cell types [7] and inhibit cancer cells’ ability to metastasize [7, 10]. Moreover, a study on breast cancer patients found that both relatively high and low vitamin D levels were associated with an unfavorable prognosis [11]. Another study found a reduced risk of advanced cancer with vitamin D supplementation [12]. There are, however, some conflicting results since another recent study found no reduced risk of any type of cancer with vitamin D supplementation [13]. In vivo and in vitro, vitamin D improved the efficacy of common cancer treatments like chemotherapy and different targeted therapies including tamoxifen [5]. This has also been shown for radiation therapy in vivo [14]. Some studies have thereto suggested that vitamin D could possibly reverse therapy resistance or at least halter it [5]. However, tumors can still develop a resistance toward Vitamin D [5]. Besides the level of vitamin D in plasma, the effect of vitamin D also depends on the interaction with the VDR and the subsequent effect on the gene expression in the cell nuclei [15].

Research regarding cancer treatments have largely focused on the genomic alterations in tumors and less on the genomic alterations or polymorphisms in patients, possibly affecting the effect and metabolization of different drugs [16, 17]. This could also be the case for vitamin D and its receptor where numerous SNPs have been identified. Yet, only a small fraction of the polymorphisms identified [18] have been extensively studied [19–21]. The present study focuses on four SNPs: Taq1 (rs731236), Tru91 (rs757343), Bsm1 (rs1544410), and Fok1 (rs2228570). These SNPs are in the beginning and in the end of the *VDR* gene in both coding and non-coding regions [22–25]. All four *VDR* SNPs are known to modulate *VDR* mRNA stability and/or expression [22–25]. Taq1 and Bsm1 have been associated with higher systemic vitamin D levels [24]. In addition, these SNPs were reported to be related to bone mineral density [25], which has mechanistically been correlated to reactivation of dormant tumor cells in bone [26, 27]. Therefore, these four *VDR* SNPs were selected as candidate SNPs in our study. Details of the effect of each *VDR* SNP are presented in Supplementary Table 1.

Several studies have investigated these SNPs in relation to breast cancer risk, prognosis, and treatment with various results [19–21, 28–32]. The Fok1 polymorphism was not associated with breast cancer risk in three studies on mostly premenopausal Caucasian and Hispanic women [19, 21, 29]. However, one study on mostly Caucasian nurses found a large association of the Fok1 polymorphism with breast cancer risk, even after adjusting for ER-status, PR-status, invasiveness, menopausal status, and vitamin D levels [20]. Regarding the Bsm1 polymorphism, three studies on Caucasian and Turkish women showed no increased risk of breast cancer [20, 21, 28]. Interestingly, the Bsm1 polymorphism has been associated with both higher and lower risk of breast cancer in a Caucasian cohort and a Hispanic cohort respectively [29, 32]. Only one study has investigated the Tru91 polymorphism and found an association with premenopausal breast cancer in Pakistani women [30]. The Taq1 polymorphism has on the other hand been properly investigated and four studies on Caucasian and Turkish women reported no association of the polymorphism with breast cancer risk [19, 21, 28, 31]. However, two of the studies found a relationship between the Taq1 polymorphism and an increased risk of ER-positive breast cancer tumors for both pre- and postmenopausal women [19, 31]. The small study on premenopausal women in Sweden also showed an association between the Taq1 polymorphism and a better prognosis with regards to lymph node metastasis and mortality [31]. The exact role and impact of the polymorphisms on the development of breast cancer is still unclear and controversial, which underline the need for future research.

The aim of this study was to investigate the associations between four *VDR* SNPs (Taq1, Tru91, Bsm1, and Fok1) and breast cancer prognosis. In addition, the study explored any potential interactions between the SNPs and important clinicopathological factors and treatment regimens that could affect the prognosis.

## Materials and methods

### Study population

The study is based on data from the BCBlood cohort in Lund, Sweden. Eligible patients included those diagnosed with primary breast cancer, without any other malignancies in the 10 years preceding their inclusion, and who had not yet undergone surgery. Details of the BC Blood cohort have previously been outlined [33]. At the time of enrollment, participants completed a three-page lifestyle questionnaire and had anthropometric measurements taken by research nurses. Body mass index (BMI) was calculated as weight in kgs divided by height in m^2^ and categorized into overweight ≥ 25 kg/m^2^ or not. After surgery at Skåne University Hospital in Lund, patients went on postoperative follow-up visits up to three years. The patients were thereafter followed using mailed out biannual follow-up questionnaires for up to 15 years postoperatively. These questionnaires collected information on medication intake, reproductive history, smoking and alcohol consumption, and types of adjuvant treatments. Clinical data regarding tumor characteristics, adjuvant treatments, and clinical outcome were gathered from medical records and registries. The ER and PR positivity cut-offs were > 10% stained nuclei as per Swedish clinical routine. Human epidermal growth factor receptor 2 (HER2) status was not incorporated into clinical routine until November 2005. HER2 status was obtained from dual gene protein staining of HER2 on tissue microarrays for patients with missing HER2 status. The method showed 97.7% agreement with available pathological assessment [34]. The study was approved by the Lund University Ethics Committee (Dnr 75-02, Dnr 37-08, Dnr 658-09, and amendments). All patients provided written informed consent.

**Fig. 1:**
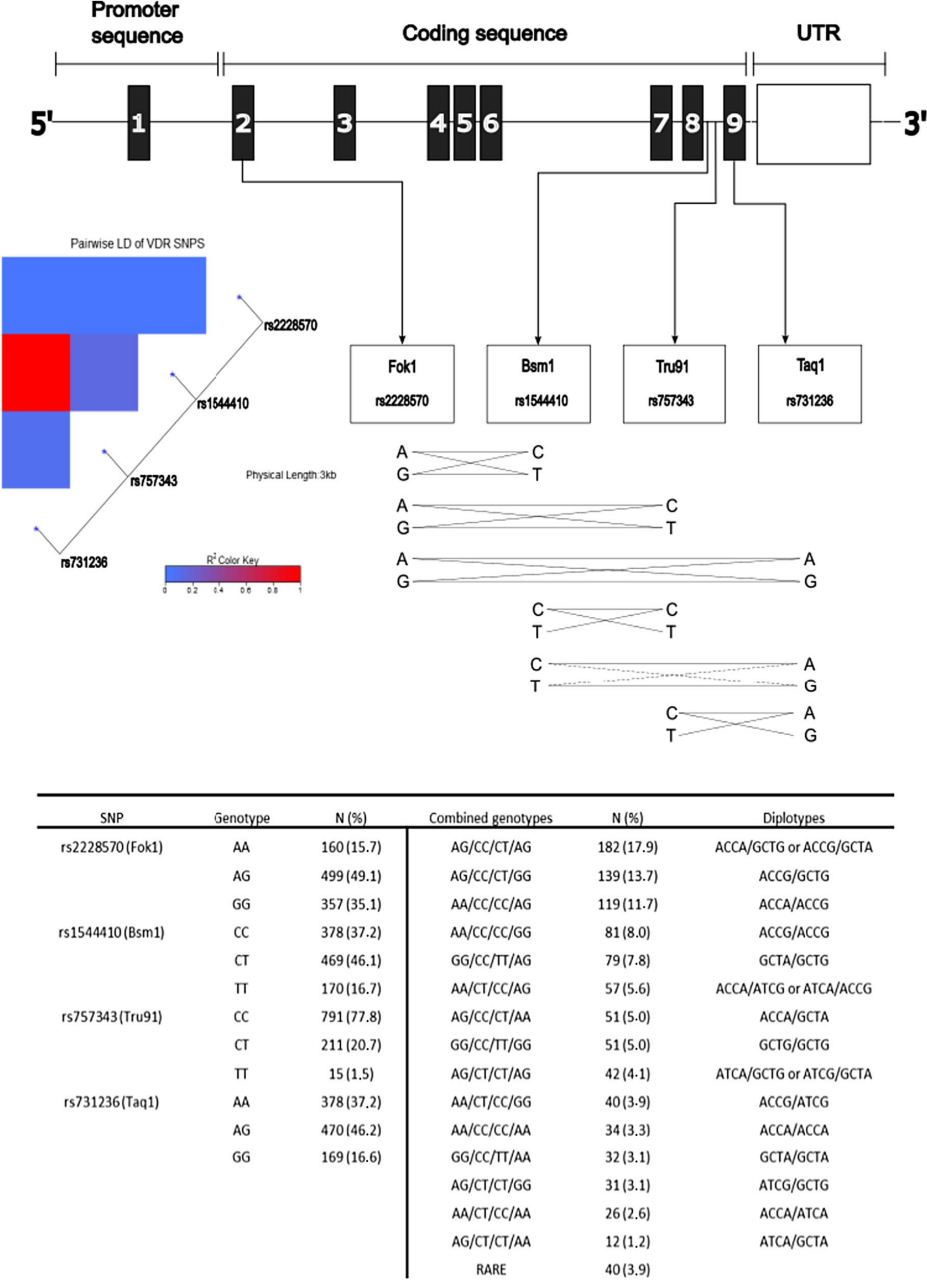
The genomic region of the *VDR* gene, accompanied by a heatmap visualizing linkage disequilibrium among the studied *VDR* SNPs. Continuous lines portray frequently observed SNP combinations, while dotted lines denote less frequent associations. The figure provides a visual summary of the inter-relationships among the SNPs within the *VDR* gene, highlighting the linkage disequilibrium that exists between certain SNPs. It also presents the frequency distribution of the *VDR* SNPs, combined genotypes and diplotypes among the 1017 breast cancer patients included in the study cohort

**Fig. 2:**
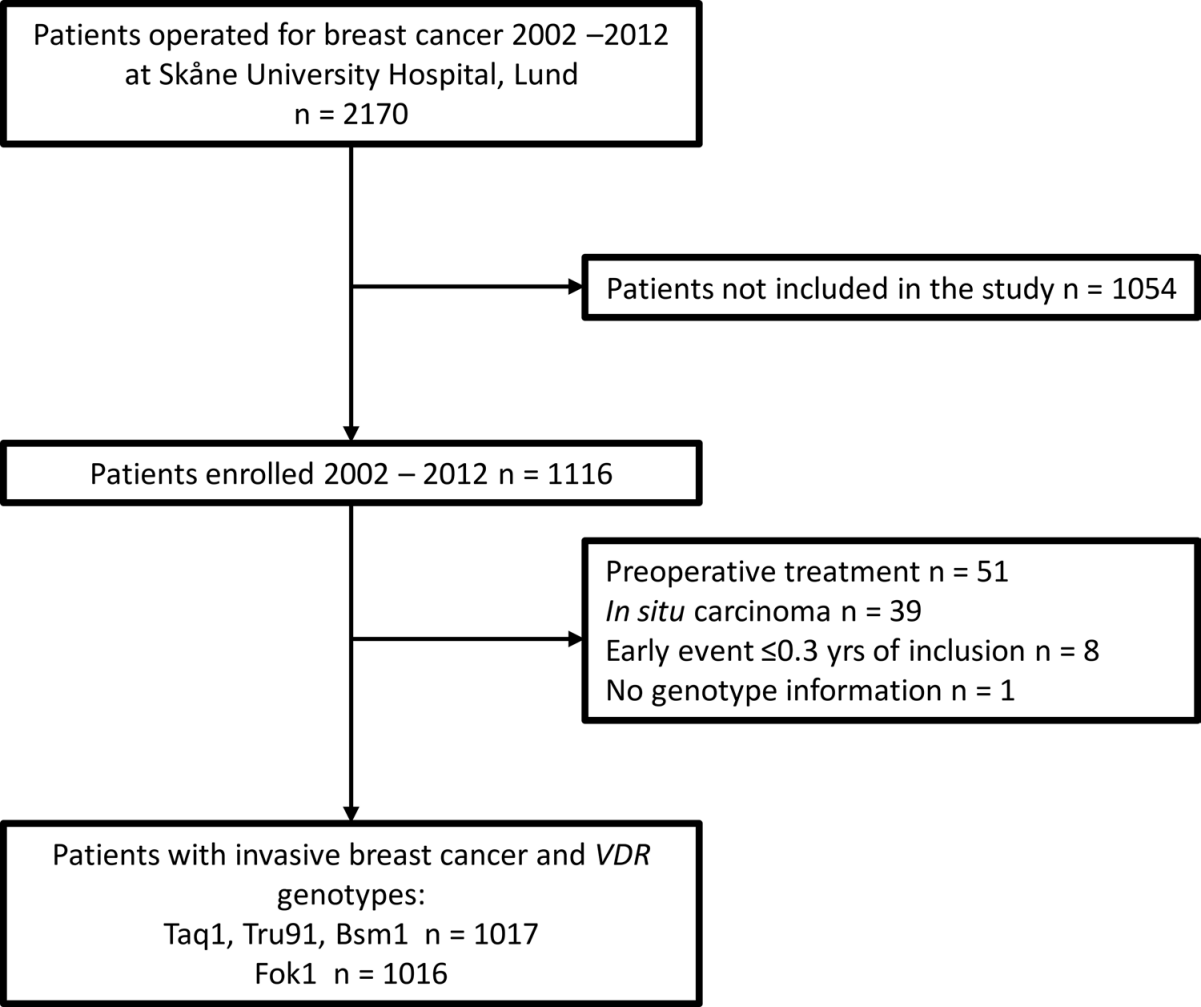
Flowchart of included and excluded patients

The patients in the present study were included from October 2002 to June 2012 and were followed until June 30, 2019. Exclusion criteria were carcinoma in situ, preoperative treatment, and distant metastasis within 0.3 years of inclusion. After, a total of 1,018 patients remained. One patient lacked genotype information entirely and was therefore excluded, leaving 1,017 patients used for analysis. Four patients lacked genotype information for Taq1, and one patient lacked genotype information for Fok1. The missing Taq1 genotype information was imputed for the four patients with missing genotype with the help of the linkage disequilibrium between Taq1 and Bsm1 visualized in Fig. 1. An overview of the selection process can be found in the flowchart, Fig. 2.

### Genotyping

*VDR* SNPs were genotyped using Oncoarray by Illumina at the Center for Translational Genomics at Lund University. The DNA used was extracted from leukocytes with DNeasy® Blood and tissue kit and processed in accordance with manufacturer’s instructions with QiaCube (Qiagen, Hilden, Germany). The Oncoarray method is customized to screen for a large number of SNPs associated with cancer [35]. All scans performed were checked according to standard quality control and low call rates rendered exclusion of the sample. SNPs with a frequency < 1% or a call rate < 99% were also excluded [36]. Forty-one SNPs in the *VDR* gene were available on the Oncoarray platform, of which six had missing data or low minor allele frequency, leaving 35 SNPs of which four candidate SNPs were selected: rs731236 Taq1, rs757343 Tru91, rs1544410 Bsm1, and rs2228570 Fok1.

### Analysis of linkage disequilibrium and haplotypes

The four *VDR* SNPs were initially cross tabulated with each other to determine the linkage disequilibrium between them. This data, coupled with statistics on the Caucasian population in Europe, were used to infer the haplotypes for each genotype in the sample. When multiple haplotype combinations were possible after the linkage disequilibrium was considered, the most plausible combination was chosen. Unfortunately, the haplotypes of the most common genotype (and some other genotypes) were not possible to determine. This made any further analysis with haplotypes impossible. The most common combined *VDR* genotype in the cohort (AG/CC/CT/AG) and homozygosity for the normal variant of all four SNPs (AA/CC/CC/AA) were instead analyzed to get some indication of the combined SNP effect. Linkage disequilibrium between VDR SNPs in the 1,000 genome project was calculated using the ‘LDLinkR’package. A heatmap was created to illustrate the linkage disequilibrium between the SNPs using ‘LDheatmap’ package in R(v4.0.2).

### Statistical analyses

Statistical analyses were conducted using SPSS version 28 (IBM, Armonk, NYC, US). Differences in clinicopathological factors, lifestyle, risk factors, and treatment depending on the genotypes of each SNP were analyzed using Chi-square test. Only patients with ER-positive tumors were analyzed when comparing differences in treatment with tamoxifen and aromatase inhibitors.

The breast cancer-free interval (BCFI) was defined as time between inclusion and the first breast cancer event, or last follow-up, *i.e.*, the period during which the patient has not experienced any recurrence of the disease. The endpoint, breast cancer event is comprised of locoregional recurrence, distant metastasis, or contralateral breast cancer. Patients without any breast cancer event were censored at time of emigration, death, or the last follow-up by June 30, 2019. Overall survival (OS) was calculated from inclusion up until death or the last follow-up until June 30, 2019.

Kaplan–Meier curves were used to illustrate the relationship between *VDR* SNPs and either BCFI or OS. For univariable survival analysis the Log-Rank-tests were used to examine associations between *VDR* SNP genotypes, the AG/CC/CT/AG (yes or no), and the AA/CC/CC/AA-genotype (yes or no) concerning BCFI and OS. Dichotomous variables were created when a clear trend was observed in the Kaplan–Meier curves between having one or two variant alleles compared with the normal variant or normal variant plus one variant allele.

Multivariable survival analysis was conducted with Cox proportional regression models to identify any associations between each of the *VDR* genotypes and BCFI or OS. The dichotomous variables created were also analyzed in the multivariable models. The models were adjusted for age at inclusion (continuous), pT2/3/4, any axillary lymph node involvement, histological grade III, ER+ , and adjuvant treatments including chemotherapy, radiation therapy, trastuzumab, tamoxifen, and aromatase inhibitors. Further, two-way multiplicative interaction analyses between each SNP genotype with the pT2/3/4, any axillary lymph node involvement, and ER+ as well as adjuvant treatments were conducted. When an interaction was discovered, the models were stratified on the respective factor and visualized in separate Kaplan–Meier curves. To investigate if a factor might be a mediator for a SNP, sensitivity analyses were conducted, where the potential mediator was omitted from the Cox models.

All *p*-values were 2-tailed and a *p*-value < 0.05 was considered statistically significant. Since this was an exploratory study, nominal p-values without adjustment for multiple testing are presented in the study [37].

## Results

### VDR genotypes in relation to clinicopathological data

Figure 1 shows the distribution of the four SNPs and any linkage between them in the study population. Notably, Bsm1 and Taq1 were in linkage disequilibrium. Heterozygous genotypes were most frequently observed for Taq1, Bsm1, and Fok1, whereas for Tru91, most patients were homozygous CC carriers. The descriptive clinicopathological data in relation genotypes are presented in Table 1. PR-negative breast cancer was more frequently observed in patients who were heterozygous for Taq1 (AG 66.8%) and/or Bsm1 (CT 66.7%) compared to homozygous Taq1 carriers (AA 74.3%, GG 75.9%) and/or Bsm1 carriers (CC 74.1%, TT 75.9%). A similar result regarding PR-negativity was seen for Fok1 (AG 67.7% *vs.* AA 74.3% and GG 74.2%). This pattern was not observed for Tru91. Additionally, lobular cancer was about twice as frequent in heterozygous carriers of Taq1 and/or Bsm1 compared to homozygous carriers. Patients carrying the Fok1 AA genotype were less likely to have large tumors (pT2/3/4) compared to AG and GG carriers (AA 19.4% vs. AG 27.7% and GG 30.5%).

**Table 1:** Descriptive statistics of the cohort regarding different lifestyle factors, treatments, and clinicopathological factors

			*VDR* Taq1 rs731236 *n* = 1017			*VDR* Tru91 rs757343 *n* = 1017			*VDR* Bsm1 rs1544410 *n* = 1017			*VDR* Fok1 rs2228570 *n* = 1016		
	All patients	Missing	AA	AG	GG	CC	CT	TT	CC	CT	TT	AA	AG	GG
	*n* = 1017		*n* = 378 (37.2%)	*n* = 470 (46.2%)	*n* = 169 (16.6%)	*n* = 791 (77.8%)	*n* = 211 (20.7%)	*n* = 15 (1.5%)	*n* = 378 (37.2%)	*n* = 469 (46.1%)	*n* = 170 (16.7%)	*n* = 160 (15.7%)	*n* = 499 (49.1%)	*n* = 357 (35.1%)
	Number (%)	Number	Number (%)	Number (%)	Number (%)	Number (%)	Number (%)	Number (%)	Number (%)	Number (%)	Number (%)	Number (%)	Number (%)	Number (%)
Age at inclusion, ≥ 50 years	815 (80.1)	0	299 (79.1)	377 (80.2)	139 (82.2)	641 (81.0)	166 (78.7)	8 (53.3)	298 (78.8)	377 (80.4)	140 (82.4)	123 (76.9)	404 (81.0)	287 (80.4)
BMI ≥ 25 kg/m^2^	502 (50.8)	28	196 (53.1)	226 (49.8)	80 (48.2)	390 (50.6)	102 (50.0)	10 (66.7)	198 (53.7)	222 (49.0)	82 (49.1)	77 (48.4)	252 (52.1)	173 (50.0)
Waist circumference ≥ 80 cm	730 (74.6)	38	277 (75.7)	330 (73.3)	123 (75.5)	565 (74.1)	153 (75.7)	12 (80.0)	278 (76.0)	326 (72.6)	126 (76.8)	108 (70.1)	360 (75.0)	262 (75.9)
Waist-hip ratio ≥ 0.85	518 (52.9)	38	211 (57.7)	226 (50.2)	81 (49.7)	397 (52.1)	111 (55.0)	10 (66.7)	209 (57.1)	227 (50.6)	82 (50.0)	81 (52.6)	249 (51.9)	188 (54.5)
Preoperative smoker, yes	206 (20.3)	2	70 (18.5)	97 (20.7)	39 (23.1)	170 (21.5)	35 (16.7)	1 (6.7)	67 (17.7)	98 (21.0)	41 (24.1)	23 (14.4)	111 (22.3)	72 (20.2)
Alcohol abstainer, yes	105 (10.4)	3	44 (11.7)	43 (9.1)	18 (10.7)	82 (10.4)	20 (9.5)	3 (20.0)	44 (11.7)	41 (8.7)	20 (11.8)	17 (10.7)	61 (12.3)	27 (7.6)
Menopausal hormone therapy, ever	445 (43.9)	3	171 (45.4)	207 (44.2)	67 (39.6)	353 (44.7)	88 (41.9)	4 (26.7)	172 (45.6)	204 (43.7)	69 (40.6)	68 (42.5)	222 (44.6)	155 (43.7)
Oral contraceptives, ever	722 (71.1)	1	274 (72.5)	331 (70.6)	117 (69.2)	562 (71.1)	150 (71.1)	10 (66.7)	275 (72.8)	329 (70.3)	118 (69.4)	117 (73.1)	355 (71.3)	250 (70.0)
Screening detected (45–74 years)	568 (66.2)	159	223 (67.6)	250 (64.6)	95 (67.4)	443 (65.8)	118 (69.0)	7 (50.0)	222 (67.5)	254 (65.5)	92 (65.2)	98 (71.0)	267 (63.3)	203 (66.8)
*Invasive tumor size*														
pT2/3/4	279 (27.4)	0	98 (25.9)	139 (29.6)	42 (24.9)	221 (27.9)	56 (26.5)	2 (13.3)	98 (25.9)	136 (29.0)	45 (26.5)	31 (19.4)	138 (27.7)	109 (30.5)
Any axillary lymph node involvement	390 (38.4)	2	158 (41.9)	169 (36.0)	63 (37.5)	295 (37.4)	88 (41.7)	7 (46.7)	159 (42.2)	167 (35.6)	64 (37.9)	57 (35.8)	189 (38.0)	143 (40.1)
*Receptor status*														
ER^+^	894 (87.9)	0	338 (89.4)	407 (86.6)	149 (88.2)	693 (87.6)	187 (88.6)	14 (93.3)	338 (89.4)	404 (86.1)	152 (89.4)	143 (89.4)	432 (86.6)	318 (89.1)
PR^+^	722 (71.0)	0	281 (74.3)	314 (66.8)	127 (75.1)	554 (70.0)	154 (73.0)	14 (93.3)	280 (74.1)	313 (66.7)	129 (75.9)	119 (74.4)	338 (67.7)	265 (74.2)
HER2 Amplification	110 (11.5)	16	37 (10.5)	55 (12.3)	18 (11.5)	79 (10.7)	29 (14.5)	2 (14.3)	37 (10.5)	56 (12.6)	17 (11.0)	13 (8.7)	54 (11.5)	42 (12.5)
Triple Negative	75 (7.4)	6	25 (6.6)	38 (8.1)	12 (7.2)	60 (7.6)	14 (6.7)	1 (6.7)	25 (6.6)	40 (8.6)	10 (6.0)	11 (6.9)	45 (9.1)	19 (5.4)
*Main histological type*														
No special type (formerly ductal)	821 (80.7)	0	317 (83.9)	362 (77.0)	142 (84.0)	630 (79.6)	178 (84.4)	13 (86.7)	319 (84.4)	360 (76.8)	142 (83.5)	138 (86.3)	395 (79.2)	287 (80.4)
Lobular	118 (11.6)	0	32 (8.5)	73 (15.5)	13 (7.7)	97 (12.3)	19 (9.0)	2 (13.3)	32 (8.5)	74 (15.8)	12 (7.1)	13 (8.1)	61 (12.2)	44 (12.3)
Other or mixed	78 (7.7)	0	29 (7.7)	35 (7.4)	14 (8.3)	64 (8.1)	14 (6.6)	0 (0.0)	27 (7.1)	35 (7.5)	16 (9.4)	9 (5.6)	43 (8.6)	26 (7.3)
Histological grade III	254 (25.0)	1	86 (22.8)	125 (26.6)	43 (25.6)	196 (24.8)	54 (25.6)	4 (26.7)	86 (22.8)	125 (26.7)	43 (25.4)	34 (21.3)	141 (28.3)	78 (21.8)
*Ever treatment by last follow-up prior to any event*														
Chemotherapy	257 (25.3)	0	99 (26.2)	122 (26.0)	36 (21.3)	192 (24.3)	59 (28.0)	6 (40.0)	99 (26.2)	123 (26.2)	35 (20.6)	39 (24.4)	129 (25.9)	88 (24.6)
Radiotherapy	644 (63.3)	0	246 (65.1)	292 (62.1)	106 (62.7)	502 (63.5)	131 (62.1)	11 (73.3)	247 (65.3)	289 (61.6)	108 (63.5)	100 (62.5)	308 (61.7)	235 (65.8)
Herceptin	72 (7.1)	0	23 (6.1)	37 (7.9)	12 (7.1)	53 (6.7)	18 (8.5)	1 (6.7)	23 (6.1)	38 (8.1)	11 (6.5)	9 (5.6)	38 (7.6)	24 (6.7)
*ER*^+^ *tumors*														
Tamoxifen	572 (64.0)	0	222 (65.7)	263 (64.6)	87 (58.4)	449 (64.8)	112 (59.9)	11 (78.6)	223 (66.0)	261 (64.6)	88 (57.9)	87 (60.8)	279 (64.6)	206 (64.8)
Aromatase inhibitor	371 (41.5)	0	155 (45.9)	156 (38.3)	60 (40.3)	292 (42.1)	74 (39.6)	5 (35.7)	156 (46.2)	151 (37.4)	64 (42.1)	58 (40.6)	187 (43.3)	125 (39.3)

### VDR genotypes in relation to prognosis

In the univariable survival analyses the most common combined genotype AG/CC/CT/AG was not associated with either BCFI or OS (Supplementary Fig. 1A, B). However, in the multivariable analyses adjusted for age, tumor characteristics, and treatments, a statistically significant association between this most common combined genotype and higher breast cancer event risk was observed (hazard ratio (HR) 1.44, 95% CI = 1.01–2.04; Supplementary Table 2). The combined genotype AA/CC/CC/AA was not associated with clinical outcome in either the univariable or multivariable analyses (Supplementary Fig. 1C, D and Supplementary Table 3).

**Fig. 3:**
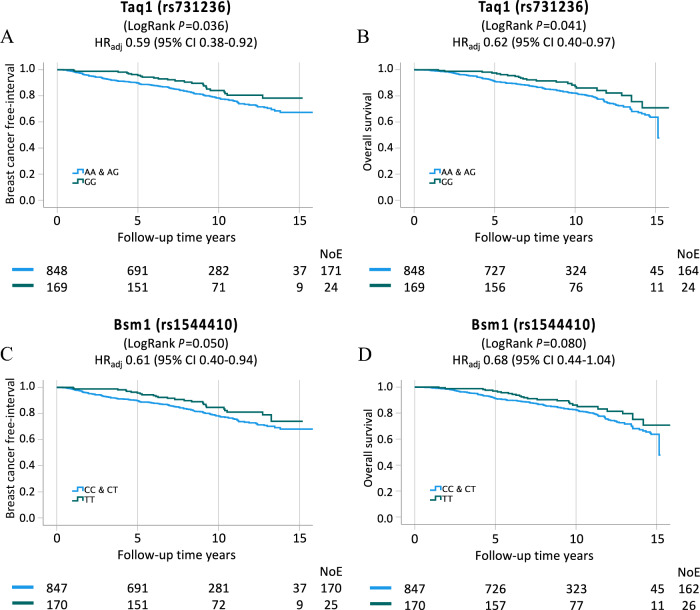
Kaplan–Meier estimates of dichotomized Taq1 (GG against combined AA and AG genotype) in relation to (A) BCFI, and (B) OS in all patients. Kaplan–Meier estimates of dichotomized Bsm1 (TT against combined CC and CT genotype) in relation to (C) BCFI, and (D) OS in all patients. Adjusted HR with 95% CI for each genotype is presented. The multivariable Cox regression models were adjusted for age, tumor characteristics and adjuvant treatments. The number of patients is indicated at each follow-up. The study is ongoing; thus, the number of patients decreases with each follow-up

There were trends of improved BCFI and OS for carriers of homozygous variants of Taq1 (GG) and Bsm1 (TT), in the univariable analysis (Supplementary Fig. 2A, D, Supplementary Table 4 and 5). The Taq1 GG genotype was associated with longer BCFI (LogRank *P* = 0.036) and OS (LogRank *P* = 0.041) compared to the AG/GG genotypes (Fig. 3A, B). The Bsm1 TT genotype was borderline associated with longer BCFI (LogRank *P* = 0.050) and OS (LogRank *P* = 0.080) compared to the CT/CC genotypes (Fig. 3C, D). In the multivariable survival analyses, carriers of the Taq1 GG genotype had a statistically significant decreased risk of breast cancer events and death compared to AG/AA genotypes, BCFI adjusted HR 0.59, 95% CI 0.38–0.92 and for OS adjusted HR 0.62, 95% CI 0.40–0.97 (Table 2A). A similar trend was observed for the Bsm1 TT genotype, with a decreased risk of breast cancer events (adjusted HR 0.61, 95% CI 0.40–0.94), but did not reach statistical significance for OS (adjusted HR 0.68, 95% CI 0.44–1.04; Table 2B).

**Table 2:** Multivariable models of Taq1 GG and Bsm1 TT genotype in relation to risk of breast cancer event and death

	Breast cancer events			Death		
	Hazard ratio	Confidence interval 95%		Hazard ratio	Confidence interval 95%	
		Lower	Upper		Lower	Upper
A) *Taq1*						
Taq1 AA/AG	Ref.	Ref.	Ref.	Ref.	Ref.	Ref.
Taq1 GG	0.59	0.38	0.92	0.62	0.40	0.97
Age at inclusion	0.99	0.98	1.01	1.05	1.04	1.07
pT2/3/4	1.96	1.42	2.71	0.61	0.36	1.05
Any axillary lymph node involvement	1.83	1.27	2.63	1.36	0.94	1.96
ER^+^	1.04	0.60	1.79	1.82	1.32	2.50
Histological grade III	1.61	1.11	2.35	1.33	0.91	1.94
Chemotherapy	0.68	0.42	1.10	1.20	0.72	1.99
Radiation therapy	0.81	0.60	1.09	0.93	0.69	1.27
Tamoxifen	0.63	0.46	0.87	0.87	0.61	1.23
Aromatase inhibitor	0.63	0.43	0.92	0.84	0.56	1.25
Trastuzumab	0.71	0.38	1.32	0.51	0.25	1.05
B) *Bsm1*						
Bsm1 CC/CT	Ref.	Ref.	Ref.	Ref.	Ref.	Ref.
Bsm1 TT	0.61	0.40	0.94	0.68	0.44	1.04
Age at inclusion	0.99	0.98	1.01	1.05	1.04	1.07
pT2/3/4	1.97	1.43	2.72	0.61	0.36	1.05
Any axillary lymph node involvement	1.82	1.27	2.62	1.36	0.95	1.96
ER^+^	1.04	0.61	1.79	1.82	1.32	2.51
Histological grade III	1.61	1.11	2.34	1.32	0.90	1.93
Chemotherapy	0.68	0.42	1.10	1.20	0.72	1.98
Radiation therapy	0.81	0.60	1.09	0.93	0.68	1.27
Tamoxifen	0.63	0.45	0.87	0.87	0.61	1.24
Aromatase inhibitor	0.63	0.43	0.93	0.84	0.57	1.26
Trastuzumab	0.70	0.38	1.32	0.51	0.25	1.05

The Tru91 genotypes were not associated with clinical outcomes in the univariable or the multivariable analyses (Supplementary Fig. 2E, F; Supplementary Table 6.

None of the Fok1 genotypes were associated with BCFI (Supplementary Fig. 2G; Supplementary Table 7). However, the heterozygous form of Fok1 was associated with decreased OS in the univariable (LogRank 2d.f. *P* = 0.014; Supplementary Fig. 2H) but not the multivariable model (Supplementary Table 7).

**Fig. 4:**
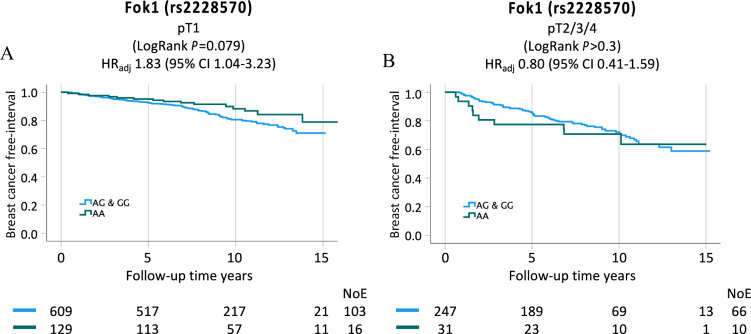
Kaplan–Meier estimates of BCFI in all patients stratified by tumor (pT1 and pT2/3/4) in relation to the dichotomous Fok1 VDR SNP (AA genotype against combined AG/GG genotype). Adjusted HR with 95% CI for each genotype is also presented. The multivariable Cox regression models were adjusted for age, tumor characteristics, and adjuvant treatments

Since Fok1 genotypes were also associated with tumor size, we excluded tumor size in sensitivity analyses to test whether the tumor size was a mediator. The effect estimates remained essentially the same, suggesting that it is not a mediator. However, there was an interaction between Fok1 GG genotype and tumor size on BCFI in the adjusted model (adjusted HR 0.38, *P*_interaction_ = 0.049). For subgroup analysis, the GG and AG genotypes were combined (GG/AG) to avoid small groups (Fig. 4A, B). In the interaction analysis with the combined AG/GG genotype, the interaction became borderline statistically significant (adjusted HR 0.43, *P*_interaction_ = 0.058; Supplementary Table 8). In the subgroup analysis, the Fok1 genotypes (GG/AG) were associated with increased risk of breast cancer events in patients with smaller tumors (pT1, HR 1.83, 95% CI 1.04–3.23; Supplementary Table 9). In contrast, in patients with larger tumors (pT2/3/4), the Fok1 genotypes (AG/GG) were not associated with BCFI (adjusted HR 0.80 95% CI 0.41–1.59; Supplementary Table 9). In terms of adjuvant treatments, there were no interactions between any of the treatments and any of the four SNPs with respect to breast cancer events or death.

## Discussion

The main finding of this study was that certain germline VDR genotypes were associated with breast cancer prognosis. This is in line with the previous study by Aristarco et al*.* [38]. The present study identified an association between the Fok1 SNP and larger tumor sizes. Specifically, individuals with the AA genotype exhibited a lower frequency of larger tumors and patients with the AG and GG genotypes demonstrated a higher frequency of larger tumors. This observation implies that the presence of a Fok1 polymorphism may increase the risk of developing larger tumors. Furthermore, the Fok1 AA genotype was found in the multivariable analyses to be associated with a better prognosis and fewer breast cancer events for patients with small tumor size. However, in the interaction analyses the Fok1 AA genotype was associated with a worse prognosis compared to the GG genotype for larger tumors. These results may be explained by the biological effects of the Fok1 SNP. The GG genotype confers an mRNA molecule with lower transcriptional activity, resulting in lower abundance of VDR and thereby a weaker antiproliferative effect, which in turn may explain the faster tumor growth and larger tumor size upon discovery. Though, this does not explain the protective effect of the GG genotype for the patients with large tumors. Maybe the fact that patients with large tumors are more likely to receive more adjuvant treatment, explains why the genotype does not associate with a worse prognosis in these patients. In contrast, patients with the same genotype but smaller tumors may still have the possible disadvantage of the genotype conferring lower abundance of VDR but do not receive the extra treatment needed. One study has also shown that VDR receptor expression is lower in larger tumors compared to smaller tumors [39]. Earlier studies on the Fok1 polymorphism have largely examined the SNP in relation to risk of breast cancer and to our knowledge, there are no other studies that have investigated Fok1 in relation to breast cancer prognosis [19–21, 29]. One large study found an association between Fok1 and overall breast cancer risk [20] while three other large studies found no increased risk of breast cancer with the Fok1 polymorphism [19, 21, 29]. But to our knowledge no other study has investigated the Fok1 polymorphism in relation to the risk of large breast cancer tumors.

The Bsm1 and Taq1 polymorphism in this study was shown to be in linkage disequilibrium and showed similar associations to breast cancer events and death. The TT genotype for Bsm1 and the GG genotype for Taq1 were associated with a longer disease-free interval and a better prognosis compared to the CC/CT and AA/AG genotypes respectively. This is in alignment with a pervious study on *BRCA1/2*-negative women with invasive breast cancer and a family history of breast cancer [38]. In addition, there was an association between the Taq1 GG genotype and Bsm1 TT genotype with a longer overall survival even though the Bsm1 association was borderline statistically significant. However, this contrasts the result in a previous study that did not find such an association [38]. The polymorphisms have both been suggested to influence mRNA stability [25]. They have also been associated with higher levels of vitamin D [24], which could explain the better prognosis. Since the Bsm1 and Taq1 polymorphisms is in linkage disequilibrium and show similar results, there may be some genomic variant close to the genomic region of Bsm1 and Taq1 that is responsible for the effects. Unfortunately, it was not possible to conduct any analysis on the haplotypes to further investigate this. However, the most common combined genotype was associated with a higher risk of breast cancer events compared to the other genotypes, although it is impossible to draw any conclusion from this since the haplotypes for the AG/CC/CT/AG was not possible to determine.

An unexpected result was the lack of an association between the *VDR* SNPs and ER status since two studies have shown that the Taq1 polymorphism was associated with a higher frequency of ER+ breast cancer [19, 31]. However, there was a statistically significant difference between PR+ breast cancer and the Taq1 and Bsm1 genotypes. The heterozygous genotypes had a lower frequency of PR+ breast cancer. PR-positivity is considered to be a downstream marker of functional ER [40]. Since there were no evidence of dominant or co-dominant effect of the variant Taq1 or Bsm1 alleles and neither an association with ER status, the findings could be due to multiple testing. By the same reasoning, the association between heterozygosity for Taq1 and Bsm1 with an increased frequency of lobular breast cancer may also be a chance finding. Alternatively, the findings are due to an unmeasured confounder.

There were no interactions between any of the *VDR* SNPs and type of adjuvant treatment, in terms of risk of breast cancer events or death in the present study. Since some of the SNPs have been associated with vitamin D levels [24], this was unexpected because vitamin D have been shown to enhance chemotherapy and target therapy in animal models and cell cultures [5].

The frequencies of the different SNP genotypes in our study were similar to the distributions in other studies based on people with European background for Taq1, Bsm1, and Fok1 [19–21] and based on Pakistani women for Tru91 [30]. The frequencies of the SNPs also match the 1,000 genomes European reference distribution in the National Library of Medicine [41–44].

A limitation to the study is that we could not determine the haplotypes for each patient in the cohort. This can be solved by investigating more SNPs. The levels of vitamin D were not available in the study, which could have affected the results since it has been shown that the level of vitamin D may be associated with prognosis [45] and also interacts with the VDR. The genotyping was conducted with the Oncoarray genotyping method, and the results were not validated with an independent method. Although the method is not approved for clinical practice, it is still of high quality and customized to screen for many SNPs associated with cancer [35]. The risk for false calls should therefore be small.

The use of surveys to acquire information may introduce a risk of recall bias and drop-out bias. However, in the BCblood cohort over 90% of the follow-up surveys have been answered by the participants [46]. The other parameters used such as waist circumference were measured by trained nurses and both the patient and the nurse do not know which genotype of the SNPs the patient has, keeping any form of bias to a minimum. Another strength of the cohort investigated is its relatively large size, consisting of 1,017 patients, with nearly full genotype information as only one patient missed the Fok1 genotype. The patients included in the study can be of any social or economic background since healthcare is heavily subsidized in Sweden. Questions regarding ethnic background were never asked, however it can be assumed that the majority of the patients were of European descent, based on the demographic distribution in the area served by the hospital. If the results are confirmed by other studies, they should be applicable to people living in Scandinavia of European descent. This is because it can be assumed that they have similar exposure to the sun and distribution of the SNP genotypes.

One strength in the statistical analysis was the use of interaction variables that made it possible to discover associations where a genotype was a risk factor in small tumors but not in large tumors. However, there are some limitations of our study, such as not adjusting for multiple testing. Statistically significant results with *P*-values close to 0.05 may be due to chance. While explorative hypothesis generating studies may not always need to adjust for multiple testing, it is often used in confirmatory studies to establish associations, or in genome wide association studies. Multiple adjustment testing can potentially discard newfound associations that warrant further investigation [37]. Only four candidate SNPs were investigated. Due to the observational nature of this study, the findings may be due to unmeasured confounders and potentially not causal. However, the results are based on multivariable models adjusted for important clinically used factors, thereby eliminating key potential confounders. Therefore, *VDR* genotypes may confer independent information beyond established prognostic factors currently used in clinical models.

## Conclusion

The Taq1 GG and Bsm1 TT *VDR* genotypes were associated with improved clinical outcome and the prognostic impact of the Fok1 AA *VDR* genotype was dependent on tumor size. If confirmed, *VDR* genotypes may be used to more refined tailoring of adjuvant breast cancer treatment. Further research is needed to confirm the findings and elucidate their potential clinical implications.

### Supplementary Information

Below is the link to the electronic supplementary material.

Supplementary file1 (PDF 1138 kb)

Supplementary file2 (PDF 173 kb)

## Data Availability

Clinical data are not publicly available due to privacy laws. Questions regarding data can be directed to the corresponding author.

## Abbreviations

BCFI: Breast cancer free interval
BMI: Body mass index
CI: Confidence interval
ER: Estrogen receptor
HER2: Human epidermal growth factor receptor 2
HR: Hazard ratio
OS: Overall survival
PR: Progesterone receptor
SNP: Single nucleotide polymorphism
VDR: Vitamin D receptor
