# Supplementary material for: Potential interplay between tumor size and vitamin D receptor (VDR) polymorphisms in breast cancer prognosis: a prospective cohort study
Source: Cancer Causes Control. 2024 Feb 14;35(6):907–19. doi: 10.1007/s10552-023-01845-1 (PMC11130020; doi:10.1007/s10552-023-01845-1)
Supplement: Supplementary file 1 — Supplementary file1 (PDF 1138 kb) [file 10552_2023_1845_MOESM1_ESM.pdf]

Supplementary Figures

Supplementary Figure 1.

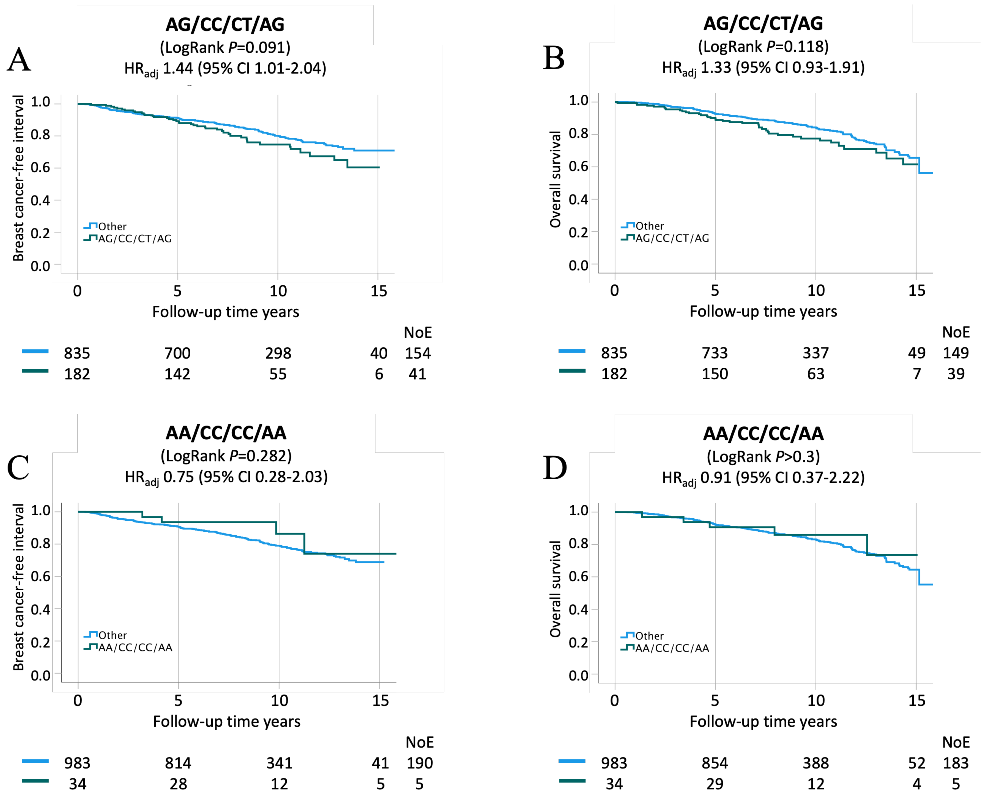

Supplementary Figure 1. Comparative survival analysis of two combined genotypes in breast cancer patients. Kaplan-Meier curves A and B illustrate the impact of the combined genotype AG/CC/CT/AG versus other genotypes in relation to BCFI and OS. Kaplan-Meier curves C and D focus on the combined genotype AA/CC/CC/AA in comparison to other genotypes, with outcomes presented in terms of BCFI in C and OS in D. Adjusted HR with 95% CI for each genotype is also presented. The multivariable Cox regression models were adjusted for age, tumor characteristics and adjuvant treatments.

Supplementary Figure 2.

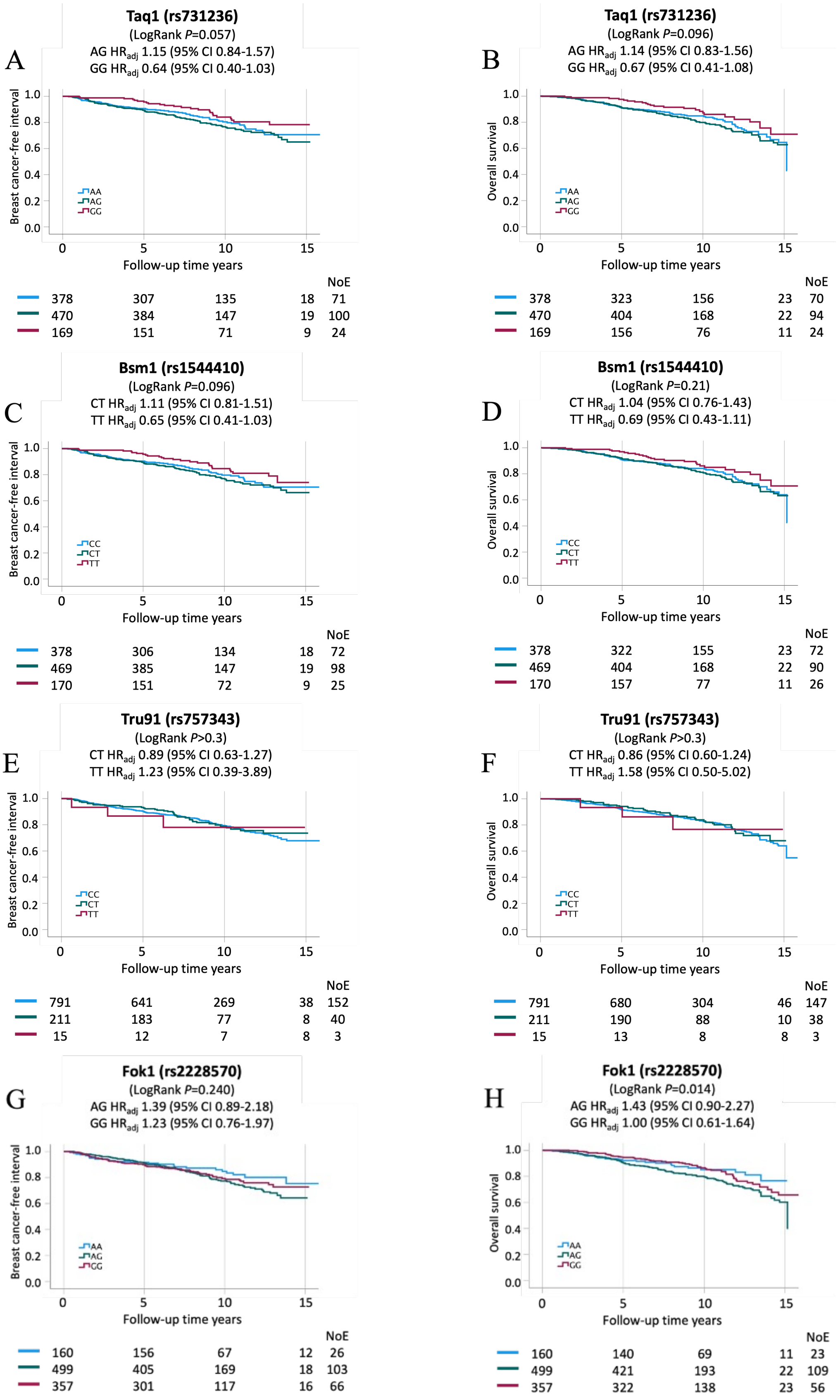

Supplementary Figure 2. Kaplan-Meier curves of Taq1 (A-B), Bsm1 (C-D), Tru91 (E-F), and Fok1 (G-H) in relation to BCFI (A, C, E, G) and OS (B, D, F, H). For each SNP, three survival curves based on the corresponding genotypes are illustrated in relation to both BCFI and OS. Adjusted HR with 95% CI for each genotype is also presented. The multivariable Cox regression models were adjusted for age, tumor characteristics and adjuvant treatments.
