# Supplementary material for: Potential interplay between tumor size and vitamin D receptor (VDR) polymorphisms in breast cancer prognosis: a prospective cohort study
Source: Cancer Causes Control. 2024 Feb 14;35(6):907–19. doi: 10.1007/s10552-023-01845-1 (PMC11130020; doi:10.1007/s10552-023-01845-1)
Supplement: Supplementary file 2 — Supplementary file2 (PDF 173 kb) [file 10552_2023_1845_MOESM2_ESM.pdf]

Supplementary Tables

Supplementary Table 1. Overview of the investigated four *VDR* SNPs and their potential effects.

SNP	Location	rs Number	Changes amino acid sequence	Potential effects on VDR function or expression
Taq1	Exon 9	rs731236	No	It has been associated with higher circulating levels of vitamin D [24] and may affect mRNA stability [25].
Tru91	Intron 8	rs757343	No	The SNP Tru91 may affect the transcription of the *VDR* gene and the stability and expression of the transcribed mRNA molecule [23].
Bsm1	Intron 8	rs1544410	No	The SNP Bsm1 may affects gene expression through its impact on the stability of the transcribed mRNA molecule [25]. It has also been associated with higher circulating levels of vitamin D [24].
Fok1	Exon 2	rs2228570	Yes	The SNP Fok1 causes an alteration in the DNA-sequence ahead of the start codon of the *VDR* gene, which results in an additional start codon. The change results in a mRNA molecule with lower translational activity and a longer VDR protein [22].

Supplementary Table 2. Multivariable model of AG/CC/CT/AG (the most common) genotype in relation to risk of breast cancer events and death

	Breast cancer events			Death		
	Hazard ratio	Confidence interval 95%		Hazard ratio	Confidence interval 95%	
		Lower	Upper		Lower	Upper
AG/CC/CT/AG	1.44	1.01	2.04	1.33	0.93	1.91
Age at inclusion	0.99	0.98	1.01	1.05	1.04	1.07
pT2/3/4	1.97	1.43	2.72	1.83	1.33	2.51
Any axillary lymph node involvement	1.88	1.30	2.72	1.39	0.97	2.01
ER positive	1.03	0.60	1.78	0.59	0.35	1.02
Histological grade III	1.56	1.07	2.28	1.26	0.86	1.85
Chemotherapy	0.69	0.42	1.12	1.21	0.73	2.00
Radiotherapy	0.81	0.61	1.09	0.92	0.68	1.26
Tamoxifen	0.63	0.46	0.88	0.87	0.61	1.23
Aromatase inhibitor	0.63	0.43	0.92	0.84	0.57	1.25
Trastuzumab	0.70	0.38	1.31	0.51	0.25	1.04

Supplementary Table 3. Multivariable model of AA/CC/CC/AA genotype in relation to risk of breast cancer events and death

	Breast cancer events			Death		
	Hazard ratio	Confidence interval 95%		Hazard ratio	Confidence interval 95%	
		Lower	Upper		Lower	Upper
AA/CC/CC/AA	0.75	0.28	2.03	0.91	0.37	2.22
Age at inclusion	0.99	0.98	1.01	1.05	1.04	1.07
pT2/3/4	1.96	1.42	2.70	1.81	1.31	2.49
Any axillary lymph node involvement	1.81	1.26	2.61	1.36	0.95	1.97
ER positive	1.04	0.60	1.79	0.60	0.35	1.03
Histological grade III	1.57	1.08	2.29	1.29	0.88	1.89
Chemotherapy	0.71	0.44	1.15	1.23	0.74	2.05
Radiotherapy	0.80	0.60	1.08	0.92	0.68	1.25
Tamoxifen	0.65	0.47	0.89	0.89	0.63	1.26
Aromatase inhibitor	0.64	0.43	0.93	0.85	0.57	1.26
Trastuzumab	0.71	0.38	1.32	0.51	0.25	1.05

Supplementary Table 4. Multivariable model of Taq1 genotypes in relation to risk of breast cancer event and death

	Breast cancer events			Death		
	Hazard ratio	Confidence interval 95%		Hazard ratio	Confidence interval 95%	
		Lower	Upper		Lower	Upper
Taq1 AA	ref.	ref.	ref.	ref.	ref.	ref.
Taq1 AG	1.15	0.84	1.57	1.14	0.83	1.56
Taq1 GG	0.64	0.40	1.03	0.67	0.41	1.08
Age at inclusion	0.99	0.98	1.01	1.05	1.04	1.07
pT2/3/4	1.95	1.41	2.69	1.80	1.31	2.49
Any axillary lymph node involvement	1.84	1.28	2.65	1.37	0.95	1.97
ER positive	1.03	0.60	1.77	0.61	0.35	1.04
Histological grade III	1.60	1.10	2.33	1.31	0.90	1.92
Chemotherapy	0.68	0.42	1.10	1.20	0.72	1.99
Radiation therapy	0.81	0.60	1.09	0.93	0.68	1.26
Tamoxifen	0.63	0.46	0.87	0.87	0.61	1.23
Aromatase inhibitor	0.64	0.43	0.94	0.85	0.57	1.26
Trastuzumab	0.69	0.37	1.29	0.50	0.24	1.03

Supplementary Table 5. Multivariable model of Bsm1 genotypes in relation to risk of breast cancer event and death

	Breast cancer events			Death		
	Hazard ratio	Confidence interval 95%		Hazard ratio	Confidence interval 95%	
		Lower	Upper		Lower	Upper
Bsm1 CC	ref.	ref.	ref.	ref.	ref.	ref.
Bsm1 CT	1.11	0.81	1.51	1.04	0.76	1.43
Bsm1 TT	0.65	0.41	1.03	0.69	0.43	1.11
Age at inclusion	0.99	0.98	1.01	1.05	1.04	1.07
pT2/3/4	1.96	1.42	2.71	1.82	1.32	2.51
Any axillary lymph node involvement	1.83	1.27	2.64	1.37	0.95	1.97
ER positive	1.03	0.60	1.77	0.61	0.36	1.05
Histological grade III	1.60	1.10	2.34	1.32	0.90	1.93
Chemotherapy	0.68	0.42	1.10	1.20	0.72	1.98
Radiation therapy	0.81	0.60	1.09	0.93	0.68	1.26
Tamoxifen	0.63	0.45	0.87	0.87	0.61	1.23
Aromatase inhibitor	0.64	0.44	0.94	0.85	0.57	1.26
Trastuzumab	0.69	0.37	1.30	0.51	0.25	1.05

Supplementary Table 6. Multivariable model of Tru91 genotypes in relation to risk of breast cancer event and death

	Breast cancer events			Death		
	Hazard ratio	Confidence interval 95%		Hazard ratio	Confidence interval 95%	
		Lower	Upper		Lower	Upper
Tru91 CC	ref.	ref.	ref.	ref.	ref.	ref.
Tru 91 CT	0.89	0.63	1.27	0.86	0.60	1.24
Tru91 TT	1.23	0.39	3.89	1.58	0.50	5.02
Age at inclusion	0.99	0.98	1.01	1.05	1.04	1.07
pT2/3/4	1.97	1.43	2.72	1.81	1.32	2.50
Any axillary lymph node involvement	1.85	1.28	2.66	1.40	0.97	2.03
ER positive	1.05	0.61	1.81	0.60	0.35	1.03
Histological grade III	1.58	1.08	2.31	1.29	0.88	1.89
Chemotherapy	0.70	0.43	1.14	1.22	0.74	2.03
Radiation therapy	0.80	0.60	1.08	0.92	0.67	1.25
Tamoxifen	0.64	0.46	0.88	0.87	0.62	1.24
Aromatase inhibitor	0.63	0.43	0.92	0.83	0.56	1.24
Trastuzumab	0.70	0.38	1.32	0.50	0.24	1.03

Supplementary Table 7. Multivariable model of Fok1 genotypes in relation to breast cancer events and death

	Breast cancer events			Death		
	Hazard ratio	Confidence interval 95%		Hazard ratio	Confidence interval 95%	
		Lower	Upper		Lower	Upper
Fok1 AA	ref.	ref.	ref.	ref.	ref.	ref.
Fok1 AG	1.39	0.89	2.18	1.43	0.90	2.27
Fok1 GG	1.23	0.76	1.97	1.00	0.61	1.64
Age at inclusion	0.99	0.98	1.01	1.05	1.03	1.07
pT2/3/4	1.95	1.41	2.70	1.86	1.35	2.56
Any axillary lymph node involvement	1.81	1.26	2.61	1.33	0.92	1.92
ER positive	1.06	0.62	1.83	0.61	0.36	1.05
Histological grade III	1.56	1.07	2.28	1.21	0.82	1.78
Chemotherapy	0.70	0.43	1.14	1.23	0.74	2.03
Radiation therapy	0.80	0.60	1.08	0.93	0.68	1.26
Tamoxifen	0.64	0.46	0.88	0.87	0.61	1.23
Aromatase inhibitor	0.63	0.43	0.93	0.85	0.57	1.26
Trastuzumab	0.72	0.39	1.36	0.54	0.26	1.10

Supplementary Table 8. Multivariable model of the interaction between pT and Fok1 AG/GG in relation to risk of breast cancer event

	P-value	Hazard ratio	Confidence interval 95%	
			Lower	Upper
pT2/3/4 & Fok1 AG/GG	0.058	0.43	0.18	1.03
Fok1 AG/GG	0.047	1.77	1.01	3.1
Age at inclusion	0.188	0.99	0.98	1.01
pT2/3/4	<.001	4.11	1.78	9.46
Any axillary lymph node involvement	<.001	1.86	1.29	2.68
ER positive	0.783	1.08	0.62	1.87
Histological grade III	0.016	1.6	1.09	2.33
Chemotherapy	0.143	0.69	0.43	1.13
Radiationtherapy	0.102	0.78	0.58	1.05
Tamoxifen	0.006	0.63	0.46	0.88
Aromatase inhibitor	0.02	0.63	0.43	0.93
Trastuzumab	0.224	0.68	0.36	1.27

Supplementary Table 9. Multivariable model of Fok1 AG/GG in relation to risk of breast cancer event in patients with pT1 tumors and pT2/3/4 tumors

	pT1 tumors			pT2/3/4 tumors		
	Hazard ratio	Confidence interval 95%		Hazard ratio	Confidence interval 95%	
		Lower	Upper		Lower	Upper
Fok1 AG/GG	1.83	1.04	3.23	0.80	0.41	1.59
Age at inclusion	0.99	0.97	1.01	0.99	0.97	1.02
Any axillary lymph node involvement	2.11	1.33	3.37	1.57	0.88	2.80
ER positive	1.25	0.59	2.62	0.90	0.35	2.35
Histological grade III	2.19	1.31	3.66	1.14	0.65	2.00
Chemotherapy	0.47	0.22	0.99	0.99	0.50	1.97
Radiation therapy	0.68	0.47	1.00	0.95	0.59	1.55
Tamoxifen	0.58	0.38	0.86	0.71	0.37	1.37
Aromatase inhibitor	0.67	0.41	1.12	0.56	0.29	1.08
Trastuzumab	0.97	0.36	2.61	0.57	0.25	1.32
